# Concomitant evaluation of cardiovascular and cerebrovascular controls via Geweke spectral causality to assess the propensity to postural syncope

**DOI:** 10.1007/s11517-023-02885-0

**Published:** 2023-07-15

**Authors:** Alberto Porta, Francesca Gelpi, Vlasta Bari, Beatrice Cairo, Beatrice De Maria, Davide Tonon, Gianluca Rossato, Luca Faes

**Affiliations:** 1 Department of Biomedical Sciences for Health, University of Milan, 20133 Milan, Italy; 2 Department of Cardiothoracic, Vascular Anesthesia and Intensive Care, IRCCS Policlinico San Donato, Via R. Morandi 30, San Donato Milanese, 20097 Milan, Italy; 3 IRCCS Istituti Clinici Scientifici Maugeri, 20138 Milan, Italy; 4 Department of Neurology, IRCCS Sacro Cuore Don Calabria Hospital, 37024 Negrar, Verona, Italy; 5 Department of Engineering, University of Palermo, 90128 Palermo, Italy

**Keywords:** Vector autoregressive model, Heart rate variability, Arterial pressure, Blood flow, Baroreflex, Cerebral autoregulation, Head-up tilt, Postural syncope

## Abstract

The evaluation of propensity to postural syncope necessitates the concomitant characterization of the cardiovascular and cerebrovascular controls and a method capable of disentangling closed loop relationships and decomposing causal links in the frequency domain. We applied Geweke spectral causality (GSC) to assess cardiovascular control from heart period and systolic arterial pressure variability and cerebrovascular regulation from mean arterial pressure and mean cerebral blood velocity variability in 13 control subjects and 13 individuals prone to develop orthostatic syncope. Analysis was made at rest in supine position and during head-up tilt at 60°, well before observing presyncope signs. Two different linear model structures were compared, namely bivariate autoregressive and bivariate dynamic adjustment classes. We found that (i) GSC markers did not depend on the model structure; (ii) the concomitant assessment of cardiovascular and cerebrovascular controls was useful for a deeper comprehension of postural disturbances; (iii) orthostatic syncope appeared to be favored by the loss of a coordinated behavior between the baroreflex feedback and mechanical feedforward pathway in the frequency band typical of the baroreflex functioning during the postural challenge, and by a weak cerebral autoregulation as revealed by the increased strength of the pressure-to-flow link in the respiratory band. GSC applied to spontaneous cardiovascular and cerebrovascular oscillations is a promising tool for describing and monitoring disturbances associated with posture modification.

**Graphical Abstract:**

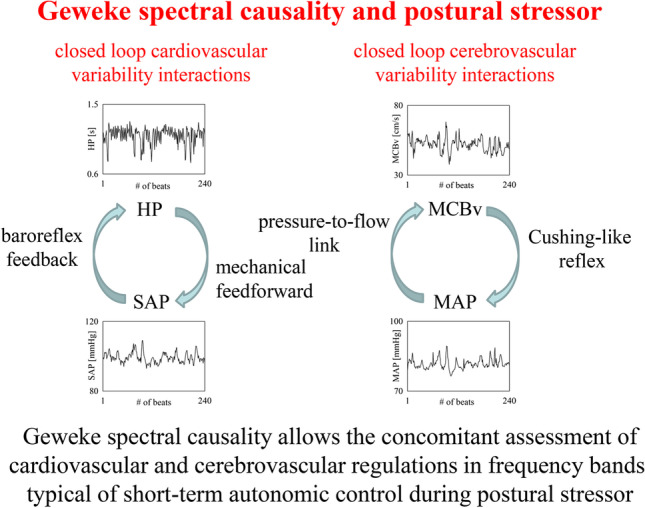

## Introduction

Regulatory mechanisms governed by the autonomic function have a profound impact on the spontaneous dynamics of cardiovascular and cerebrovascular variables and their relationships. Heart period (HP) is mainly under vagal control because atropine dramatically decreases HP variability [1], while sympathetic activation induced by an orthostatic stressor increases the magnitude of the systolic arterial pressure (SAP) fluctuations especially in the low frequency (LF) band [2, 3]. Sympathetic drive affects cerebrovascular circulation as well, even though the contribution of vagal circuits cannot be dismissed [4, 5]. Autonomic function influences the cardiac arm of the baroreflex, usually described by the dynamic relationship from SAP to HP [2, 6, 7], and cerebral autoregulation (CA), as inferred from the dynamic link from mean arterial pressure (MAP) to mean cerebral blood velocity (MCBv) [8, 9].

Model-based causality analysis [10, 11] applied to cardiovascular and cerebrovascular variability series is becoming a traditional tool in characterizing regulatory mechanisms [12, 13] aiming at stabilizing physiological variables such as arterial pressure (AP) and cerebral blood flow (CBF). Such an interest is linked to the ability of causality tools to quantify modifications of the strength of the causal link between two signals, one taken as a drive and the other as a target, occurring in response to a stimulus challenging cardiovascular and/or cerebrovascular homeostasis [14–23]. For example, the application of model-based causality analysis to cardiovascular variables suggests that orthostatic challenge increases the strength of the causal relationship from SAP to HP along the cardiac arm of the baroreflex in proportion to the magnitude of the stressor [15, 18, 24]. Model-based causality tools have been applied to describe cerebrovascular control as well: indeed, it was found that an orthostatic stressor increases the strength of the dependence of MCBv on MAP along the pressure-to-flow pathway only in individuals prone to develop postural syncope [19] thus suggesting the inability of the CA in limiting the variability of MCBv in response to MAP changes.

Some methodological key features of causality analysis are currently under scrutiny because they might have a profound impact on conclusions. First, it is still unclear whether model-based spectral causality analysis [25–33] provides additional insight compared to causal markers derived in time domain using Granger causality and/or transfer entropy [14–24]. Time domain causality approaches do not provide scale-specific information [14–24]. Conversely, physiological mechanisms operate in specific frequency bands: for example, baroreflex generates oscillations about its resonance frequency (i.e., about 0.1 Hz) [34, 35] and cerebrovascular mechanisms feature time scales even slower (i.e., from 0.02 to 0.07 Hz) [36]. Since spectral causality indexes could be made scale-specific by computing them in assigned frequency bands, those markers should be superior in describing the inherent functioning of physiological mechanisms compared to time domain causality indexes. Second, it is unclear whether the underlying model structure adopted to interpret causal relationships might have an impact on the conclusions. Model structures capable of describing rhythms that cannot be fully explained by dynamical interactions among signals, such as the dynamic adjustment model (DA) class, might provide some advantage with respect to model structures that do not have this possibility, such as the autoregressive model (AR) class [15, 27, 37, 38]. Third, it is unclear whether the concomitant analysis of cardiovascular and cerebrovascular variability interactions via spectral causality analysis might provide useful information in stratifying the risk of syncope in patients prone to orthostatic intolerance during an orthostatic stressor.

The present study is specifically designed to address the three above-mentioned issues over a historical database utilized to assess cardiovascular and cerebrovascular variability interactions in subjects prone to develop postural syncope (SYNC) compared to control individuals who never experienced postural syncope (noSYNC) [19, 39, 40]. Geweke spectral causality (GSC) [11] was utilized to differentiate cardiovascular and cerebrovascular regulations in SYNC and noSYNC groups over variability series of SAP and HP and of MAP and MCBv using two different model structures, namely the bivariate AR (BAR) and the bivariate DA (BDA) classes. We hypothesize that GSC could provide additional information compared to a previous time domain causality analysis working regardless of time scales and applied to the same data [19], and that conclusions do not depend on the structure of the model utilized to describe dynamic interactions, namely BAR or BDA.

## Experimental protocol and data analysis

### Compliance with ethical standards

The study adhered to the principles of the Declaration of Helsinki for medical research involving humans. The protocol was approved by the ethical committee of the IRCCS Sacro Cuore Don Calabria Hospital, Negrar, Italy (protocol number: 101/2010; date of approval: 1/5/2010). All subjects provided written informed consent before entering the study.

### Experimental protocol

Data were collected to explore the possibility of stratifying the risk of fainting via the analysis of spontaneous cardiovascular and cerebrovascular oscillations evoked by volume shift toward lower limbs induced by a postural stimulus [19, 39, 40]. In this study, we considered 13 SYNC subjects (age: 28 ± 9 years; 5 males) and 13 noSYNC control subjects (age: 27 ± 8 years; 5 males). The SYNC subjects were highly likely to faint because they had a history of syncope with more than 3 events/year in their last 2 years. This propensity was verified via prolonged head-up tilt protocol. Events were considered of unknown etiology because clinical and physical assessment excluded common causes linked to neurological, electrocardiographic, and hemodynamic factors. The health condition of noSYNC individuals was verified via similar clinical screening and the prolonged head-up tilt test certified their resistance to syncope. Preparation of the subjects, characteristics of the facilities where experimental sessions took place, and instructions given to the subjects were reported in [39, 40]. SYNC and noSYNC individuals were free from any medication known to interfere with cardiovascular and/or cerebrovascular controls. We acquired the electrocardiogram (ECG) in a lead II configuration, volume-clamp continuous AP from the middle finger of the right hand (Finapres Medical Systems, Enschede, The Netherlands) and CB velocity (CBv) from the right or left middle cerebral artery via a transcranial Doppler device (Multi-Dop T, DWL, 2 MHz, Compumedics, San Juan Capistrano, CA, USA) [41]. CBv was considered a proxy of CBF under the hypothesis of negligible modifications of the vessel diameter [36]. All signals were sampled synchronously at 1000 Hz. Attention was paid to leave a period of stabilization of the physiological variables before starting the acquisition session. ECG, AP and CBv were recorded for 10 min with the subject lying at rest in horizontal position (REST) on the tilt table. Then, it was rotated to reach an inclination of 60° and recording session during head-up tilt (HUT) started. Tilt table rotated continuously from horizontal position to 60° in 15 s. If no signs of presyncope were observed during HUT, the duration of the session was prolonged up to 40 min. Otherwise, the subject was immediately returned to REST. As expected, presyncope signs were observed in all SYNC subjects at different timings, while HUT session lasted 40 min in noSYNC subjects. The syncope occurred after 1047 ± 546 s from the HUT onset in SYNC subjects.

### Beat-to-beat variability series

The time interval between two consecutive R-wave peaks of the ECG was taken as the *n*th HP (HP_*n*_). The maximum AP within HP_*n*_ was identified as the *n*th systolic AP (SAP_*n*_). The *n*th diastolic arterial pressure (DAP_*n*_) was the minimum of AP following SAP_*n*_. Detections of R-wave peaks from the ECG and AP fiducial points were visually checked. Procedures to insert missed identifications, to correct and reinsert eventual misdetections and to limit the effect of arrhythmic beats were described in [39, 40]. Only few isolated ectopic beats were detected, and their number was always less than 5% of the total length of the sequence. The MAP_*n*_ was derived as the ratio of the definite integral of AP between the timing of DAP_*n*‒1_ and DAP_*n*_ to the interdiastolic interval. MCBv_*n*_ was calculated similarly using the same fiducial points for the computation of the definite integral over the CBv signal [40]. As the focus of the study was the characterization of short-term regulatory mechanisms, the analysis was carried out over sequences of 256 consecutive synchronous HP, SAP, MAP and MCBv values taken in a random position within REST and HUT sessions [36, 42]. Stationarity of mean and variance was checked as proposed in [43]. Transitory adjustments of the variables during the transition from REST to HUT were avoided. Regardless of the group, the selection of HUT segments was carried out in the early phase of HUT (i.e., within the first 10 min) and before the observation of any presyncope sign in SYNC individuals. Time domain markers were reported in [44]. Briefly, HP and MCBv means decreased during HUT in both groups. HP variance declined during HUT compared to REST in SYNC group. At REST, MAP mean was smaller in SYNC compared to noSYNC and in SYNC increased during HUT compared to REST. SAP mean and SAP, MAP and MCBv variances did not change across groups and conditions.

### Computation of GSC markers

The joint interactions between two stochastic processes ${Y}_{1}$ and ${Y}_{2}$ were described by the bivariate process $${\varvec{Y}}={\left[\begin{array}{cc}{Y}_{1}& {Y}_{2}\end{array}\right]}^{\mathrm{T}}$$, where ^T^ is the transposition operator. ${\varvec{Y}}$ belongs to the class of BAR and BDA processes [27, 38]. BAR and BDA processes differ for the structure of the innovation $${\varvec{W}}={\left[\begin{array}{cc}{W}_{1}& {W}_{2}\end{array}\right]}^{\mathrm{T}}$$ whose components are Gaussian white noises in the case of BAR process and Gaussian AR noises in the case of BDA process [27, 38]. GSC evaluates the strength of the causal interactions from ${Y}_{i}$ to ${Y}_{j}$ with *i,j* = 1,2 and *i* ≠ *j* as a function of the frequency *f* as the negative logarithm of the fractional contribution of the partial power spectral density of ${Y}_{j}$ due ${W}_{j}$ to the overall power spectral density of ${Y}_{j}$ [11]. Indeed, this fractional contribution decreases toward 0, while increasing the importance of the pathway from ${Y}_{i}$ to ${Y}_{j}$ [11]. We refer to [45] for the complete derivation of GSC markers from partial power spectral densities estimated via BAR and BDA model coefficients.

GSC markers were computed with ${Y}_{1}=\mathrm{HP}$ and ${Y}_{2}=\mathrm{SAP}$ to describe the cardiovascular regulation and with ${Y}_{1}=\mathrm{MCBv}$ and ${Y}_{2}=\mathrm{MAP}$ to describe the cerebrovascular control. Analyses were carried out over normalized series with zero mean and unit variance. More specifically, normalization was carried out by estimating a linear regression over time in the original series and by subtracting the linear trend from the original data. Each sample of the resulting zero mean series was divided by the standard deviation to obtain a series with unit variance. Coefficients of the BAR model were identified via traditional least squares technique, while those of the BDA one via generalized least squares method [38]. Identification problem was solved via the Cholesky decomposition method. Generalized least squares method was stopped [38] if the absolute value of the fractional between-iteration decline of prediction error variance was below 0.001. The model order was optimized via the Akaike information criterion for multivariate processes [46] in the range from 7 to 12. The latency from SAP to HP was set to 0 beats and that from HP to SAP to 1 beat [24, 27]. The latency from MAP to MCBv was set to 2 beats and that from MCBv to MAP to 0 beats [23, 47]. Markers were computed by integrating the GSC functions over frequency bands [11] set according to the functioning of the cardiovascular and cerebrovascular controls. For the analysis of HP-SAP variability interactions, we adopted the typical frequency bands utilized for the analysis of cardiovascular variability series [6, 42], namely the LF band from 0.04 to 0.15 Hz and the high frequency (HF) band from 0.15 to 0.4 Hz. For the analysis of MCBv-MAP variability interactions, we adopted the frequency bands utilized for the analysis of cerebrovascular variability series [36], namely the very low frequency (VLF) band from 0.02 to 0.07 Hz, LF band from 0.07 to 0.15 Hz and HF band from 0.15 to 0.4 Hz. The superior cut-off of the LF band and the inferior cut-off of the HF band were reduced to 0.15 Hz from the original suggestion [36] due to the possible presence of slow breathing rate in young individuals [47]. The GSC markers computed over HP and SAP in the LF band were denoted as GSC_SAP⟶HP_(LF) and GSC_HP⟶SAP_(LF), while those in the HF band as GSC_SAP⟶HP_(HF) and GSC_HP⟶SAP_(HF). The GSC markers computed over MCBv and MAP in the VLF band were denoted as GSC_MAP⟶MCBv_(VLF) and GSC_MCBv⟶MAP_(VLF), those in the LF band as GSC_MAP⟶MCBv_(LF) and GSC_MCBv⟶MAP_(LF), and those in the HF band as GSC_MAP⟶MCBv_(HF) and GSC_MCBv⟶MAP_(HF).

### Testing the appropriateness of the model structure

We tested the null hypothesis that the model structure was appropriate by checking whether autocorrelation computed over the model residuals and cross-correlation computed between the residuals were negligible at lags different from 0 (LAG ≠ 0) and whether cross-correlation between the residuals was insignificant at lag equal to 0 (LAG = 0). We exploited two portmanteau tests applied at LAG = 0 and LAG ≠ 0, respectively. The tests were based on the observation that the distribution of the square root of *N* times the normalized autocorrelation, or cross-correlation, converges toward a standard Gaussian distribution. More specifically, in LAG = 0 test the null hypothesis of negligible cross-correlation at lag equal to zero was rejected if the absolute value of the normalized cross-correlation at zero lag was larger than a threshold set as 1.96 divided by square root of *N* − *p*_*o*_, where *p*_*o*_ is the optimal model order. The acceptance of the null hypothesis indicated the suitability of the model structure at zero lag. In the LAG ≠ 0 test the null hypothesis of negligible autocorrelation, or cross-correlation, at lags different from 0 was rejected if the absolute value of the normalized autocorrelation, or cross-correlation, at lags different from 0 was larger than a threshold set as 1.96 divided by square root of *N* − *p*_*o*_ − *l*, where *l* was the lag. The null hypothesis of uncorrelation was rejected if the number of lags at which the absolute values of autocorrelation, or cross-correlation, was found to be above the threshold was larger than 2, being 2 the 5% of the total amount of computed autocorrelation, or cross-correlation, samples (i.e., with 1 ≤ *l* ≤ *L* with *L* = 40). The test was carried out separately over autocorrelation and cross-correlation. The acceptance of the null hypothesis indicated that both autocorrelation and cross-correlation remained limited, thus suggesting the suitability of the model structure at lags different from 0.

### Testing the null hypothesis of uncoupling via isodistribution isospectral surrogates

We tested the null hypothesis of full uncoupling [13] between the two processes ${Y}_{1}$ and ${Y}_{2}$ via the construction of sets of surrogate pairs preserving the distribution and power spectral density of the original series [48] but being fully uncoupled [49]. This null hypothesis was tested by generating 100 realizations of ${Y}_{1}$ and ${Y}_{2}$ for each original pair in any given group and experimental condition. The surrogate series were built to preserve the distribution and power spectral density of the original series, while phases were substituted with uniformly distributed random numbers ranging from 0 to 2π. We exploited an iteratively refined amplitude-adjusted, Fourier transform–based procedure to generate surrogate pairs [48]. It allowed the exact preservation of the original distribution, while the power spectrum was the best approximation of the initial power spectrum given 100 iterates. The use of two independent random phase sequences allowed the generation of fully uncoupled pairs [49]. The length of the series (i.e., 256) allowed us to speed up the construction of surrogate set via fast Fourier transform procedure. GSC markers were computed over each set of surrogates and the 95th percentile was extracted. If the marker computed over the original series was above the 95th percentile of the GSC markers derived from surrogates, the null hypothesis of uncoupling was rejected and the alternative hypothesis, namely the series were significantly associated in the considered time direction, was accepted. The percentage of subjects with a significant HP-SAP coupling was monitored as well as the one with a significant MCBv-MAP coupling. These percentages were computed for each frequency band and labelled as GSC_SAP⟶HP_(LF)%, GSC_HP⟶SAP_(LF)%, GSC_SAP⟶HP_(HF)%, GSC_HP⟶SAP_(HF)%, GSC_MAP⟶MCBv_(VLF)%, GSC_MCBv⟶MAP_(VLF)%, GSC_MAP⟶MCBv_(LF)%, GSC_MCBv⟶MAP_(LF)%, GSC_MAP⟶MCBv_(HF)% and GSC_MCBv⟶MAP_(HF)%.

### Statistical analysis

Two-way repeated measures analysis of variance (one factor repetition, Holm-Sidak test for multiple comparisons) was utilized to assess the significance of the differences between groups within the same experimental condition (i.e., REST or HUT) and between experimental conditions within the same group (i.e., SYNC or noSYNC). If hypotheses for the utilization of Holm-Sidak test were not fulfilled, nonparametric tests (i.e., Mann–Whitney rank sum and Wilcoxon signed rank tests) were applied when appropriate. The level of significance of each test was lowered according to the number of comparisons (i.e., 4) to account for the multiple comparison issue. The χ2 test (McNemar’s test) was applied to the proportion of subjects featuring the rejection of the null hypothesis of uncoupling to assess the effect of HUT within the assigned group. Traditional χ2 test was applied to check the different behavior of noSYNC and SYNC subjects within the same experimental condition. Even in this case the level of significance of the test was lowered according to the number of comparisons (i.e., 4) to account for the multiple comparison issue. The analysis over the proportion of subjects featuring the rejection of the null hypothesis of uncoupling was carried out after pooling together the results of surrogate data analysis relevant to BAR and BDA models. Statistical analysis was performed with a commercial statistical software (Sigmaplot v.14.0, Systat Software, San Jose, CA, USA). The level of statistical significance of all the tests was set to 0.05. A type-I error probability *p* smaller than the level of significance was always taken as significant.

## Results

### Results on cardiovascular control

The optimal model order *p*_*o*_ of the BAR and BDA structures describing HP-SAP dynamic interactions was reported in Table 1. *p*_*o*_ did not vary across groups and experimental conditions. The null hypothesis of negligible correlation between the residuals of HP and SAP at zero lag, tested via the LAG = 0 test, was accepted in 100% of the subjects and this result held regardless of the model structure, experimental condition, and group. The null hypothesis of insignificant autocorrelation and cross-correlation within and between the residuals at lags different from 0, tested via the LAG ≠ 0 test, was accepted in more than, or equal to, 69% of the subjects and this result held regardless of the model structure, experimental condition, and group (Table 2).

**Table 1:** Optimal model order *p*_*o*_ of BAR and BDA models describing the HP-SAP dynamical interactions

*p*_*o*_	Experimental condition	SYNC	noSYNC
BAR	REST	8.1 ± 1.6	7.8 ± 1.2
	HUT	7.6 ± 0.9	8.3 ± 1.8
BDA	REST	7.7 ± 1.4	7.2 ± 0.6
	HUT	7.7 ± 1.4	7.8 ± 1.5

*p*_*o*_, optimal model order; *SYNC*, subjects with a history of recurrent postural syncope; *noSYNC*, subjects without a history of recurrent postural syncope; *HP*, heart period; *SAP*, systolic arterial pressure; *BAR*, bivariate autoregressive model; *BDA*, bivariate dynamic adjustment model; *REST*, at rest in supine position; *HUT*, head-up tilt at 60°

**Table 2:** Percentage of acceptance of the null hypothesis of negligible autocorrelation and cross-correlation computed at lags different from 0 (LAG ≠ 0) over the residuals of the BAR and BDA models describing the HP-SAP dynamical interactions

LAG ≠ 0	Experimental condition	SYNC	noSYNC
BAR	REST	69	85
	HUT	77	77
BDA	REST	85	77
	HUT	92	92

*SYNC*, subjects with a history of recurrent postural syncope; *noSYNC*, subjects without a history of recurrent postural syncope; *HP*, heart period; *SAP*, systolic arterial pressure; *BAR*, bivariate autoregressive model; *BDA*, bivariate dynamic adjustment model; *REST*, at rest in supine position; *HUT*, head-up tilt at 60°

**Fig. 1:**
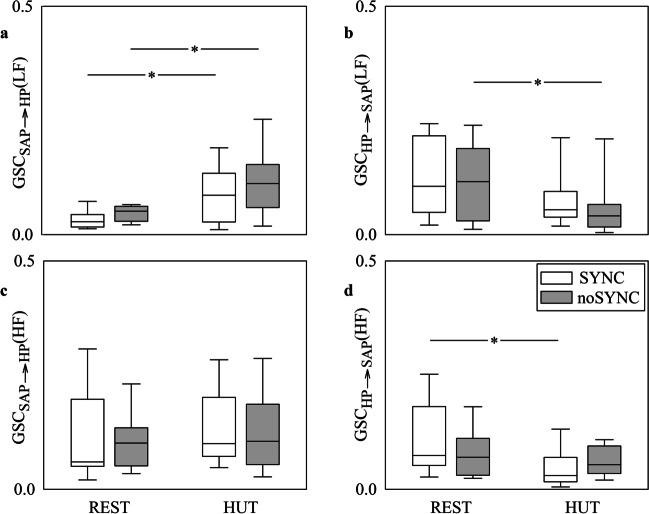
The grouped vertical box-and-whisker plots show GSC_SAP⟶HP_(LF) (**a**), GSC_HP⟶SAP_(LF) (**b**), GSC_SAP⟶HP_(HF) (**c**) and GSC_HP⟶SAP_(HF) (**d**) as a function of the experimental condition (i.e., REST and HUT). Indexes are computed in SYNC (white boxes) and noSYNC (grey boxes) subjects via the BAR model

The grouped vertical box-and-whisker plots of Fig. 1 show GSC_SAP⟶HP_(LF) (Fig. 1a), GSC_HP⟶SAP_(LF) (Fig. 1b), GSC_SAP⟶HP_(HF) (Fig. 1c) and GSC_HP⟶SAP_(HF) (Fig. 1d) as a function of the experimental condition (i.e., REST and HUT) in SYNC individuals (white boxes) and noSYNC subjects (grey boxes). Markers were computed via the BAR model. The height of the box represents the distance between the first and third quartiles, with the median marked as a line, and the whiskers show the 5th and 95th percentiles. GSC_SAP⟶HP_(LF) increased during HUT in both SYNC and noSYNC groups, while no between-group difference was detected both at REST and during HUT (Fig. 1a). GSC_HP⟶SAP_(LF) declined with HUT, but the decrease was significant only in noSYNC group, while it did not vary across groups either at REST or during HUT (Fig. 1b). The trends of GSC_HP⟶SAP_(HF) (Fig. 1d) were like those of GSC_HP⟶SAP_(LF) (Fig. 1b) but the decline with HUT was significant solely in the SYNC group. GSC_SAP⟶HP_(HF) did not vary across either experimental conditions or groups (Fig. 1c).

**Fig. 2:**
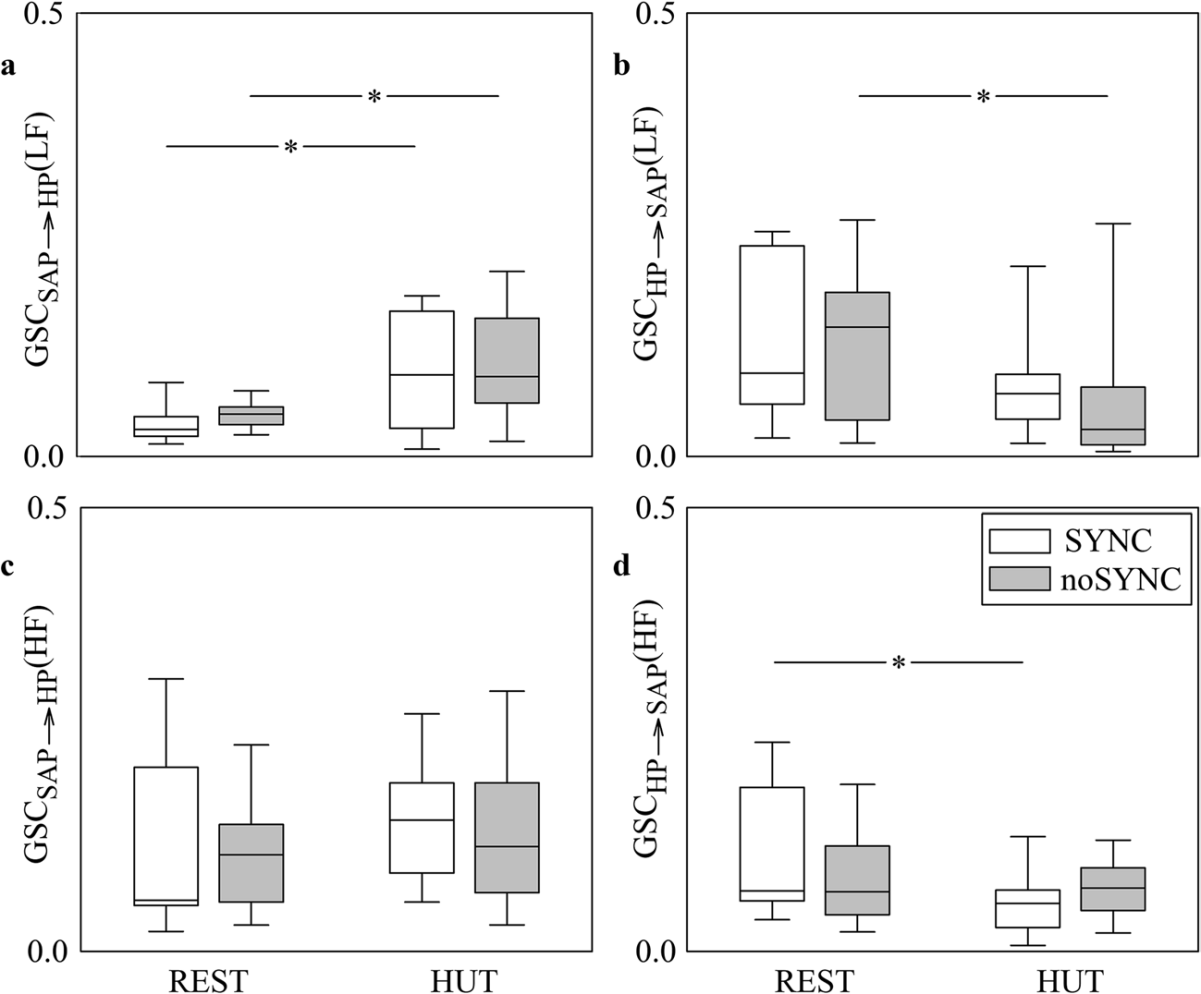
The grouped vertical box-and-whisker plots show GSC_SAP⟶HP_(LF) (**a**), GSC_HP⟶SAP_(LF) (**b**), GSC_SAP⟶HP_(HF) (**c**) and GSC_HP⟶SAP_(HF) (**d**) as a function of the experimental condition (i.e., REST and HUT). Indexes are computed in SYNC (white boxes) and noSYNC (grey boxes) subjects via the BDA model

Figure 2 has the same structure and shows the same indexes as Fig. 1, but the markers were computed via the BDA model. Trends with groups and experimental conditions are similar to those reported in Fig. 1 as well as the significance, thus suggesting that BAR and BDA models lead to the same conclusions.

**Fig. 3:**
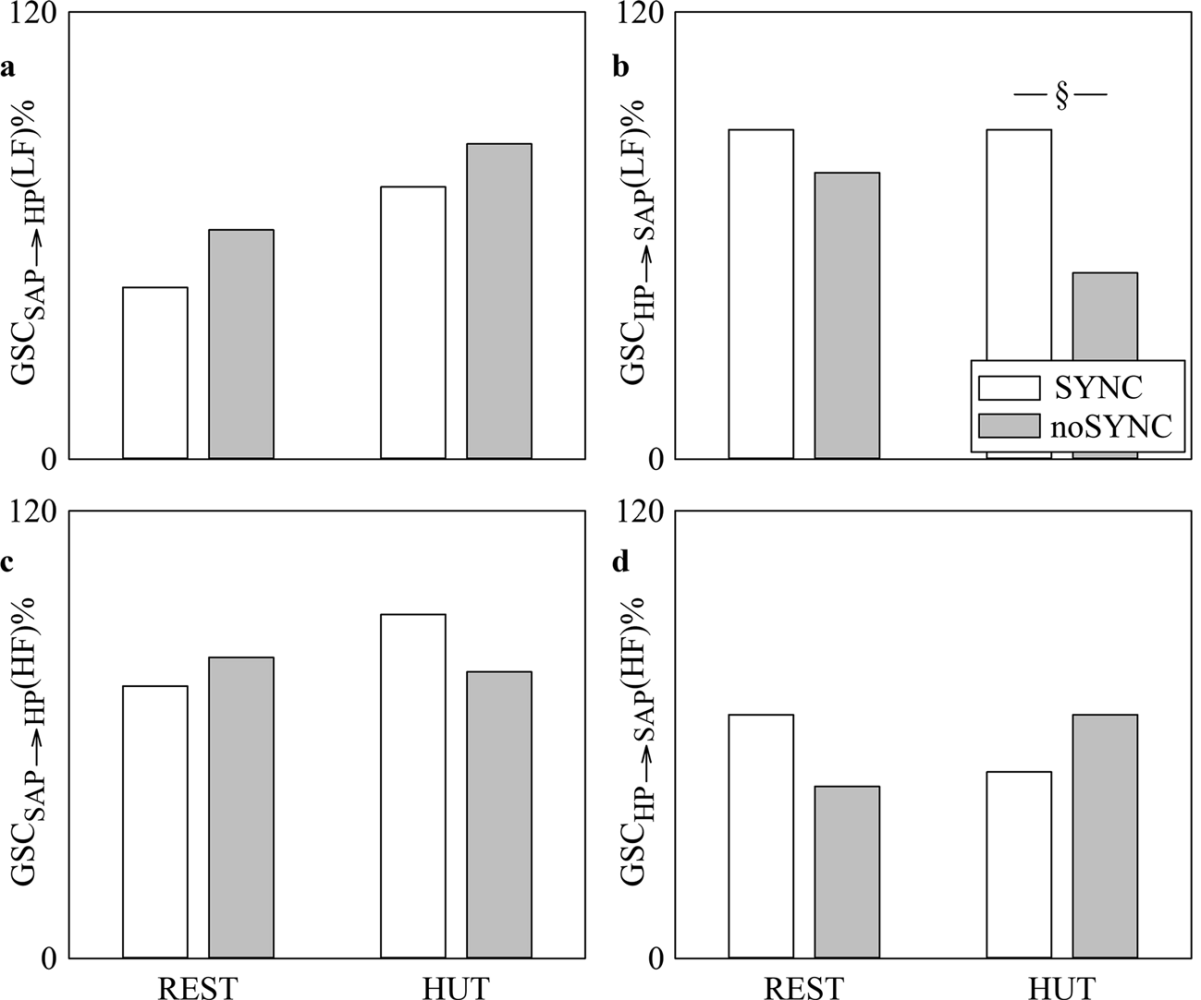
The grouped vertical bar graphs show GSC_SAP⟶HP_(LF)% (**a**), GSC_HP⟶SAP_(LF)% (**b**), GSC_SAP⟶HP_(HF)% (**c**) and GSC_HP⟶SAP_(HF)% (**d**) as a function of the experimental condition (i.e., REST and HUT). Indexes are computed in SYNC (white bars) and noSYNC (grey bars) subjects. Percentages are computed by pooling together markers derived from both BAR and BDA models

The grouped bar graphs of Fig. 3 show GSC_SAP⟶HP_(LF)% (Fig. 3a), GSC_HP⟶SAP_(LF)% (Fig. 3b), GSC_SAP⟶HP_(HF)% (Fig. 3c) and GSC_HP⟶SAP_(HF)% (Fig. 3d) as a function of the experimental condition (i.e., REST and HUT) in SYNC individuals (white bars) and noSYNC subjects (grey bars). The percentages were computed after pooling together the results of surrogate test based on BAR and BDA models given their similar performance as suggested by Fig. 1,2. GSC_SAP⟶HP_(LF)%, GSC_SAP⟶HP_(HF)% and GSC_HP⟶SAP_(HF)% did not vary across either experimental conditions or groups (Fig. 3a,c,d). GSC_HP⟶SAP_(LF)% decreased significantly in noSYNC compared to SYNC group solely during HUT, while the effect of HUT was not visible in either SYNC or noSYNC individuals (Fig. 3b).

### Results on cerebrovascular control

Table 3 shows the optimal model order *p*_*o*_ of the BAR and BDA structures describing MCBv-MAP dynamic interactions. *p*_*o*_ was not affected by HUT within the same group and remained unvaried in SYNC compared to noSYNC within the same experimental condition. The null hypothesis of negligible correlation between the residuals of MCBv and MAP at zero lag, tested via the LAG = 0 test, was accepted in 100% of the subjects and this result held regardless of the model structure, experimental condition, and group. The null hypothesis of insignificant autocorrelation and cross-correlation within and between the residuals at lags different from 0, tested via the LAG ≠ 0 test, was accepted in more than, or equal to, 62% of the subjects and this result held regardless of the model structure, experimental condition, and group (Table 4).

**Table 3:** Optimal model order *p*_*o*_ of BAR and BDA models describing the MCBv-MAP dynamical interactions

*p*_*o*_	Experimental condition	SYNC	noSYNC
BAR	REST	8.2 ± 1.7	8.3 ± 1.4
	HUT	7.7 ± 1.2	8.7 ± 1.6
BDA	REST	7.7 ± 1.3	7.9 ± 1.4
	HUT	7.6 ± 1.5	8.5 ± 1.9

*p*_*o*_, optimal model order; *SYNC*, subjects with a history of recurrent postural syncope; *noSYNC*, subjects without a history of recurrent postural syncope; *MCBv*, mean cerebral blood velocity; *MAP*, mean arterial pressure; *BAR*, bivariate autoregressive model; *BDA*, bivariate dynamic adjustment model; *REST*, at rest in supine position; HUT, head-up tilt at 60°

**Table 4:** Percentage of acceptance of the null hypothesis of negligible autocorrelation and cross-correlation computed at lags different from 0 (LAG ≠ 0) over the residuals of the BAR and BDA models describing the MCBv-MAP dynamical interactions

LAG ≠ 0	Experimental condition	SYNC	noSYNC
BAR	REST	62	85
	HUT	62	69
BDA	REST	85	85
	HUT	69	92

*SYNC*, subjects with a history of recurrent postural syncope; *noSYNC*, subjects without a history of recurrent postural syncope; *MCBv*, mean cerebral blood velocity; *MAP*, mean arterial pressure; *BAR*, bivariate autoregressive model; *BDA*, bivariate dynamic adjustment model; *REST*, at rest in supine position; *HUT*, head-up tilt at 60°

**Fig. 4:**
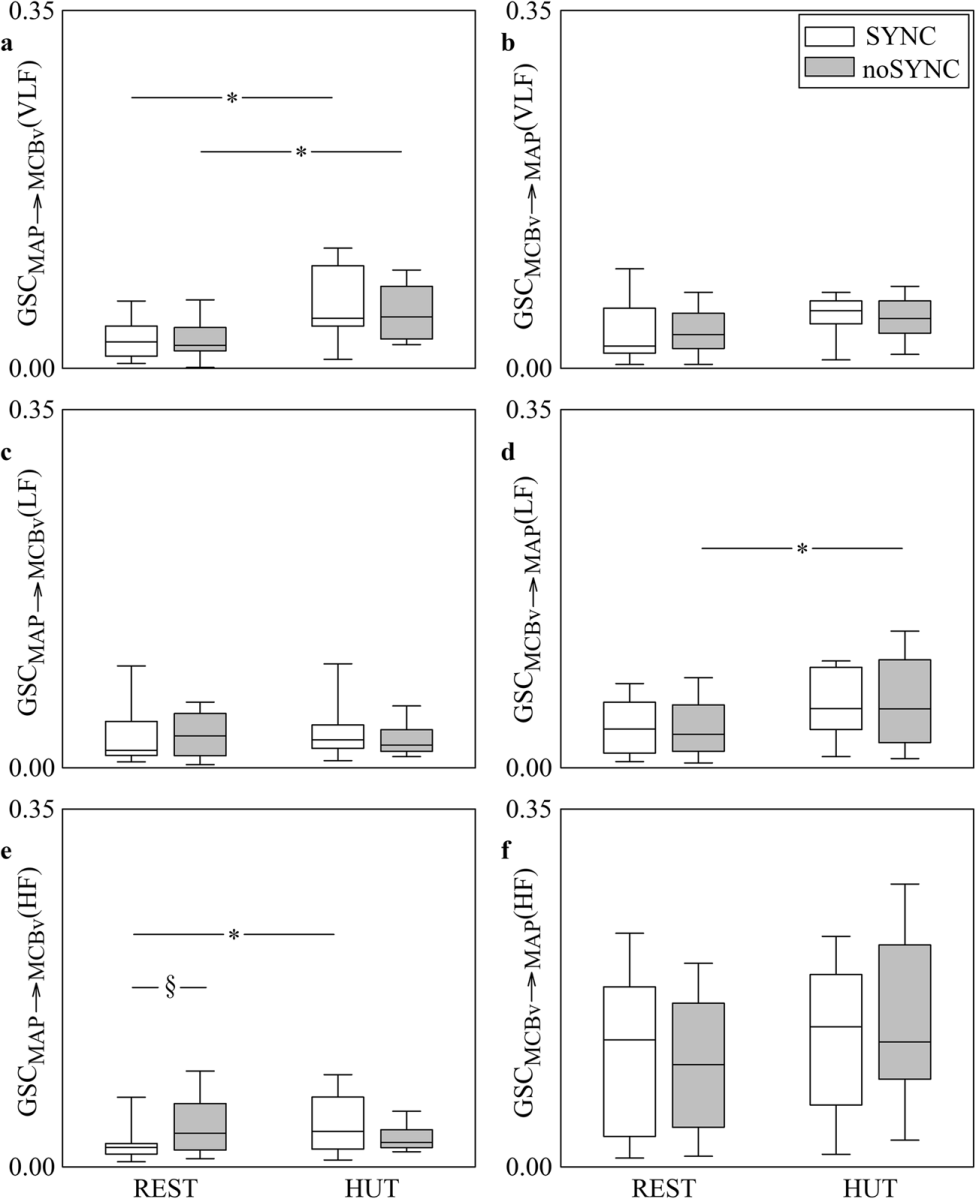
The grouped vertical box-and-whisker plots show GSC_MAP⟶MCBv_(VLF) (**a**), GSC_MCBv⟶MAP_(VLF) (**b**), GSC_MAP⟶MCBv_(LF) (**c**), GSC_MCBv⟶MAP_(LF) (**d**), GSC_MAP⟶MCBv_(HF) (**e**) and GSC_MCBv⟶MAP_(HF) (**f**) as a function of the experimental condition (i.e., REST and HUT). Indexes are computed in SYNC (white boxes) and noSYNC (grey boxes) subjects via the BAR model

The grouped vertical box-and-whisker plots of Fig. 4 show GSC_MAP⟶MCBv_(VLF) (Fig. 4a), GSC_MCBv⟶MAP_(VLF) (Fig. 4b), GSC_MAP⟶MCBv_(LF) (Fig. 4c), GSC_MCBv⟶MAP_(LF) (Fig. 4d), GSC_MAP⟶MCBv_(HF) (Fig. 4e) and GSC_MCBv⟶MAP_(HF) (Fig. 4f) as a function of the experimental condition (i.e., REST and HUT) in SYNC individuals (white boxes) and noSYNC subjects (grey boxes). Markers were computed via the BAR model. The format of any box-and-whisker plot is the same as those in Fig. 1. GSC_MAP⟶MCBv_(VLF) increased during HUT in both SYNC and noSYNC groups, while no between-group differences were detected both at REST and during HUT (Fig. 4a). GSC_MCBv⟶MAP_(LF) increased during HUT compared to REST solely in noSYNC and the two groups were similar regardless of the experimental condition (Fig. 4d). At REST GSC_MAP⟶MCBv_(HF) was higher in noSYNC compared to SYNC individuals, while it was similar in the two groups during HUT (Fig. 4e). Postural challenge raised GSC_MAP⟶MCBv_(HF) solely in SYNC subjects (Fig. 4e). GSC_MCBv⟶MAP_(VLF), GSC_MAP⟶MCBv_(LF) and GSC_MCBv⟶MAP_(HF) did not vary across either experimental conditions or groups (Fig. 4b,c,f).

**Fig. 5:**
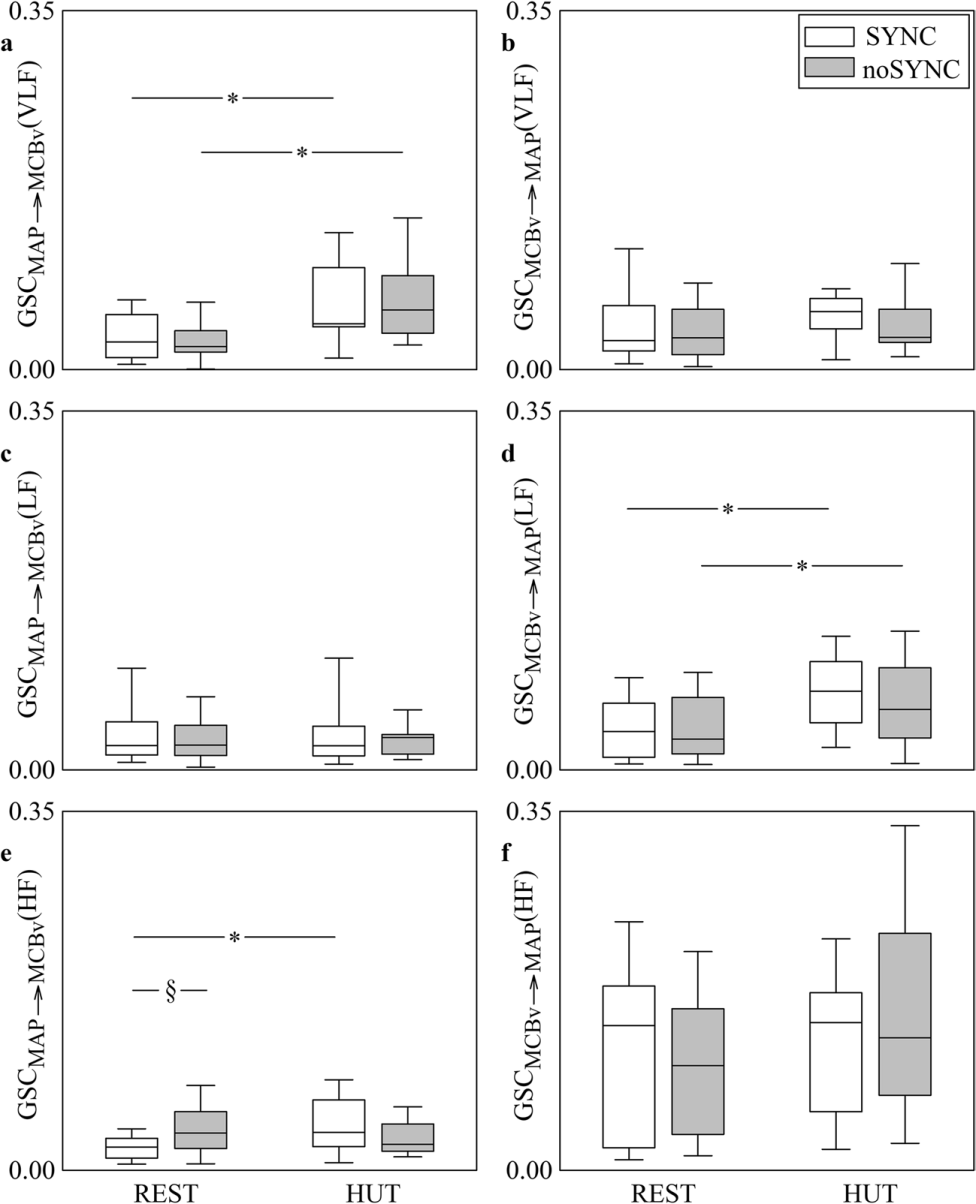
The grouped vertical box-and-whisker plots show GSC_MAP⟶MCBv_(VLF) (**a**), GSC_MCBv⟶MAP_(VLF) (**b**), GSC_MAP⟶MCBv_(LF) (**c**), GSC_MCBv⟶MAP_(LF) (**d**), GSC_MAP⟶MCBv_(HF) (**e**) and GSC_MCBv⟶MAP_(HF) (**f**) as a function of the experimental condition (i.e., REST and HUT). Indexes are computed in SYNC (white boxes) and noSYNC (grey boxes) subjects via the BDA model

Figure 5 has the same structure and shows the same indexes as Fig. 4, but the markers were computed via the BDA model. Trends with groups and experimental conditions are similar to those reported in Fig. 4. The sole additional significance compared to Fig. 4 is the increase of GSC_MCBv⟶MAP_(LF) during HUT in SYNC group (Fig. 5d). Comparison between Fig. 4 and Fig. 5 suggests that BAR and BDA models lead to the same conclusions.

**Fig. 6:**
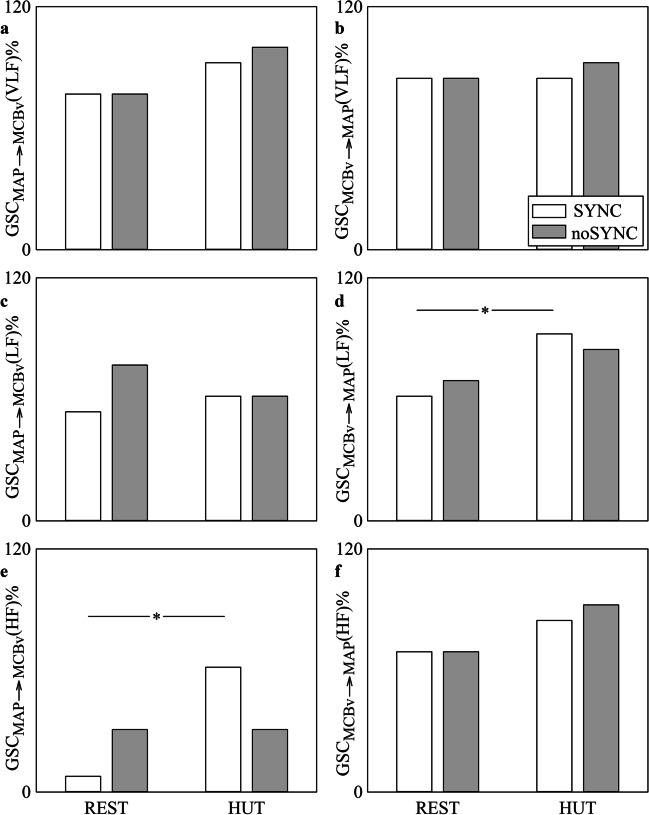
The grouped vertical bar graphs show GSC_MAP⟶MCBv_(VLF)% (**a**), GSC_MCBv⟶MAP_(VLF)% (**b**), GSC_MAP⟶MCBv_(LF)% (**c**), GSC_MCBv⟶MAP_(LF)% (**d**), GSC_MAP⟶MCBv_(HF)% (**e**) and GSC_MCBv⟶MAP_(HF)% (**f**) as a function of the experimental condition (i.e., REST and HUT). Indexes are computed in SYNC (white bars) and noSYNC (grey bars) subjects. Percentages are computed by pooling together markers derived from both BAR and BDA models

The grouped bar graphs of Fig. 6 show GSC_MAP⟶MCBv_(VLF)%, GSC_MCBv⟶MAP_(VLF)%, GSC_MAP⟶MCBv_(LF)%, GSC_MCBv⟶MAP_(LF)%, GSC_MAP⟶MCBv_(HF)% and GSC_MCBv⟶MAP_(HF)% as a function of the experimental condition (i.e., REST and HUT) in SYNC individuals (white bars) and noSYNC subjects (grey bars). The percentages were computed after pooling together the results of surrogate test based on BAR and BDA models given their similar performance as suggested by Figs. 4,5. GSC_MAP⟶MCBv_(VLF)%, GSC_MCBv⟶MAP_(VLF)%, GSC_MAP⟶MCBv_(LF)% and GSC_MCBv⟶MAP_(HF)% did not vary across either experimental conditions or groups (Fig. 6a,b,c,f). GSC_MCBv⟶MAP_(LF)% and GSC_MAP⟶MCBv_(HF)% increased during HUT solely in SYNC but they did not separate groups either at REST or during HUT (Fig. 6d,e).

## Discussion

The main findings of the study can be summarized as follows: (i) since the GSC approach can decompose the causal relationship in the frequency domain, it is particularly suitable for describing the frequency-dependent behavior of cardiovascular and cerebrovascular control mechanisms; (ii) tests carried out over residuals indicate that the model structures are suitable to describe dynamic interactions and results of the GSC analysis do not depend on the structure of the model; (iii) the development of syncope in SYNC group appear to be favored by the loss of coordinated behavior between baroreflex feedback and mechanical feedforward pathway in the frequency band typical of the AP regulation (i.e., the LF band) more than by a derangement of the baroreflex; (iv) SYNC group features a weaker CA compared to noSYNC subjects as revealed by the increased MCBv-MAP association along the pressure-to-flow link in the HF band during HUT; (v) taking all the findings together we suggest that the concomitant assessment of cardiovascular and cerebrovascular controls via a GSC approach can provide a more complete picture of the complexity of the physiological mechanisms leading to postural syncope.

### GSC provides a frequency domain decomposition of the strength of the cardiovascular and cerebrovascular causal links

The most important feature of the GSC analysis is the possibility of decomposing the strength of the causal link into contributions at given frequencies that, when summed up, provide the overall contribution in a frequency band. This feature is particularly important in cardiovascular and cerebrovascular regulations given that physiological control mechanisms are frequency-dependent.

At the level of cardiovascular control, the sinus node transfer function has a low pass filter characteristic with a much lower corner frequency when fed by fluctuations of sympathetic tone than vagal activity [50], thus leading to heart rate fluctuations with negligible contribution of the sympathetic control above 0.15 Hz and concomitant contributions of both branches of the autonomic nervous system (ANS) below 0.15 Hz [1]. The transfer function from respiration to heart rate has different properties according to the state of the ANS with a steeper role-off in situation of sympathetic overactivity and a wider bandwidth in supine resting condition [51]. Baroreflex, namely the pathway from SAP to HP, comprises a fast vagal arm and a slower branch, more under sympathetic regulation [52]. The bandwidth of the baroreflex depends on the ANS state being much more limited in situations of sympathetic activation [7]. The functioning of the link from HP to SAP varies with frequency as well, given that it lumps together Windkessel effect and Frank-Starling law [34] that depend on frequency-optimized neural controls of peripheral resistances and ventricular contractility [53, 54].

Even at the level of the cerebrovascular control, the frequency-dependent behavior of the MCBv-MAP relationship is well-known. Indeed, the link from MAP to MCBv was usually modelled as a high-pass filter in the range of frequencies of cerebrovascular variability (i.e., from 0.02 to 0.3 Hz) [55]. The autonomic control importantly modifies the frequency response of this filter. In fact, along the pressure-to-flow link ganglionic blockade increased the MCBv-MAP transfer function gain and reduced phase lead of MCBv to MAP variations [8], thus suggesting the important contribution of sympathetic control to CA. The frequency-dependent role of sympathetic regulation on cerebrovascular variability was also suggested by the increase of the MCBv-MAP transfer function gain above 0.05 Hz after α-adrenergic blockade [4]. Remarkably, this influence is extended to the reverse causal directions as well, namely to the flow-to-pressure link, commonly referred to as Cushing reflex [56], given that α1-adrenergic blockade reduced the strength of the association from MCBv to MAP [21]. However, it is worth stressing that the frequency-dependent characteristic of cerebrovascular regulation is not the exclusive consequence of sympathetic control given the important influence of the cholinergic modulation on the MCBv-MAP transfer function above 0.04 Hz [5].

### On the suitability and interchangeability of BAR and BDA model structures

We compared two linear regression models, namely BAR and BDA, widely utilized in the field of the evaluation of cardiovascular and cerebrovascular controls [7, 15, 19, 21, 23, 27, 32, 34, 35, 38, 57–64]. The two models differ for the description of the disturbance corrupting the target signal [15, 27, 37, 38]: in the BAR model the disturbing influence is modelled as a white noise, while in the BDA model, it is an AR process. Therefore, BDA is usually preferred when it could be hypothesized that some oscillatory mechanisms of unknown origin act on the target without being explained by either self- or cross-dependencies [15, 27, 37, 38], such as the activity of the respiratory centers driving HP via direct modulation of vagal outflow [65] or movements of cerebrospinal fluid driven by respiratory modulations of intrathoracic pressure capable of modifying MCBv even in the presence of stable MAP via changes of intracranial pressure [66]. Both the model structures are suitable for describing cardiovascular and cerebrovascular dynamic interactions given that the hypotheses of whiteness of the residuals and their uncorrelation, even at zero lag, was fulfilled in most of the subjects. In addition, we found that results of GSC did not depend on the structure of the model. This finding suggests that BAR is sufficient for the purpose of describing spectral contents of the causal relationships in the context of bivariate analysis. However, this result might not hold for a multivariate approach.

### SYNC group features a loss of coordinated behavior between the baroreflex and mechanical feedforward pathway in the LF band

Previous studies exploiting causality indexes operating over the entire range of time scales suggested that cardiovascular control in SYNC and noSYNC subjects exhibited different responses to HUT [19]. Indeed, while the strength of the causal relationship from SAP to HP along baroreflex increased in noSYNC individuals [15, 18, 24], postural stressor did not activate the baroreflex in SYNC subjects [19], thus suggesting an impairment of the cardiac arm of baroreflex in this group that might be mediated by a more prolonged latency [67]. The present study makes more specific the original observation reported in [19]. Indeed, the increased strength of the dependence of HP on SAP along baroreflex during HUT was observed solely in the LF band, thus confirming that baroreflex control operates more importantly in this frequency band [6, 34, 35]. An effect of the HUT over the mechanical feedforward pathway was evident as well: it took the form of a decreased strength of the link from HP to SAP in the LF band, thus suggesting the decreased importance of this pathway with respect to the baroreflex one during HUT [24]. Remarkably, this effect over the mechanical feedforward link was significant only in noSYNC individuals, thus suggesting the balance between increased strength along the baroreflex and the decreased coupling along the mechanical feedforward is important to present postural intolerance. In the HF band, HUT did not affect the degree of association from SAP to HP and marginally influenced the one on the reverse causal relationship. Our analysis could detect between-group differences as well. Remarkably, the two groups were not separated at the level of the involvement of baroreflex in governing causal dynamic interactions from SAP to HP, as it could be expected whether SYNC group exhibited a baroreflex impairment as suggested in [19]. Conversely, the relevant role of the mechanical feedforward pathway was stressed during HUT by the lower percentage of noSYNC individuals with a significant degree of coupling from HP to SAP in the LF band compared to the SYNC ones. This differentiation between the two population was not obtained using time domain casualty methods in [19], thus suggesting GSC might be more powerful in typifying the derangement of cardiovascular control than time domain causality markers. Indeed, we interpret the limited responsiveness of the baroreflex to the postural challenge observed in SYNC group detected in [19] as the result of much greater constancy of the strength from HP to SAP in the LF band preventing the identification of the activation of baroreflex in this population.

### SYNC group features a weaker CA in the HF band

The analysis of cerebrovascular control in SYNC and noSYNC subjects via causality markers that operate over the entire range of time scales suggested that the two groups exhibited different responses to HUT [19]. Indeed, while the strength of the causal relationship from MAP to MCBv remained unvaried during HUT in noSYNC individuals, it increased in SYNC individuals [19]. This finding was interpreted as a sign of the impairment of CA in the SYNC group because a working CA should limit the association between MAP and MCBv variability such a way to prevent MAP modifications to drive MCBv changes [9, 19, 68]. This study confirmed this finding and made it much more specific due to the ability of the present approach to separate frequency bands. Indeed, the strength of the pressure-to-flow dependence increased during HUT only in SYNC group in the HF band, leading to an augmentation of the percentage of SYNC subjects with a significant coupling from MAP to MCBv. The weaker level of association from MAP to MCBv at REST in SYNC group compared to noSYNC subjects is central for the detection of the significant increase during HUT in SYNC subjects, thus stressing that the healthy degree of MAP-MCBv coupling should not necessarily be too low or fully insignificant. Remarkably, SYNC and noSYNC groups exhibited different strengths of the causal link from MAP to MCBv just at REST, thus allowing us to hypothesize a structural difference of the cerebrovascular control between the two populations. It is worth noting that this result was reached in the absence of any difference between variability of MAP and MCBv and similar values of CA markers [44]. This peculiar result is evident along the pressure-to-flow link solely in the HF band: indeed, the strength of the pressure-to-flow dependence remained unvaried across groups and experimental conditions in the LF band and did not exhibit between-group difference in the VLF band. This finding might support the relevance of respiration [23] in checking the propensity to faint in SYNC group. The relevance of assessing the pressure-to-flow relationship in the HF band stresses the need to standardize tests to verify the ability of CA to limit the degree of association between MAP and MCBv in correspondence of a forcing input such as respiration. The effect of HUT was evident along the flow-to-pressure link as well. Remarkably, it was more evident in the LF band in agreement with the observation that this pathway is under sympathetic control [21].

### On the relevance of the concomitant assessment of cardiovascular and cerebrovascular controls via a GSC approach

The most striking result of the simultaneous analysis of cardiovascular and cerebrovascular controls is that the differentiation between the two populations occurred at different time scales. While at the level of the cardiovascular variability interactions markers in the LF band during HUT led to the separation of the SYNC from noSYNC group, at the level of cerebrovascular variability the between-group disjunction was achieved in the HF band at REST. This finding leads us to hypothesize that respiratory input in control condition might be sufficient to unveil the impairment at the level of cerebrovascular control, while testing the efficiency of cardiovascular regulation requires a challenge empowering LF oscillations of baroreflex origin such as HUT. The present study stresses the critical role of the missed coordination between mechanical feedforward pathway and baroreflex feedback in cardiovascular control in the frequency band including the resonance frequency of the baroreflex loop (i.e., 0.1 Hz) and indicates the possible contribution of a dysregulated MCBv response to respiratory fluctuations of MAP as factors promoting the susceptibility of SYNC group to postural failure. These findings support the conclusion that the sole assessment of either cardiovascular or cerebrovascular control might be insufficient to probe the complexity of the physiological regulations because it could provide a limited view on the system functioning. In addition, since this conclusion held only in specific frequency bands, being different in cardiovascular and cerebrovascular dynamic interactions, the application of a GSC method becomes mandatory because causality approaches in time domain working regardless of time scales cannot be useful at this regard.

The concomitant assessment of cardiovascular and cerebrovascular controls might be particularly relevant in studying postural intolerance disturbances. Postural syncope is an extremely debilitating problem experienced by subjects with a derangement of the autonomic control and even by healthy individuals after bed rest or spaceflight. Postural syncope occurs in response to a cerebral hypoperfusion secondary to a systemic hemodynamic failure. While the final event is the drop of cerebral perfusion, it is unclear whether an impairment of CA could contribute to syncope by exacerbating a CBF decline associated to a critical hemodynamic status or by even triggering a degeneration of an original hemodynamic situation that per se would not lead to hypoperfusion and consequent loss of consciousness. One of the factors that might contribute to a reduced brain perfusion during orthostatic challenge is the sympathetic activation induced by a change of posture [2, 3, 69, 70]: indeed, vasoconstriction at peripheral level, aiming at preserving venous return and cardiac output, reduces CBv at the level of brain circulation [9, 44, 71–73]. The contemporaneous analysis of cardiovascular and cerebrovascular controls might provide the correct perspective to elucidate the temporal sequence of events leading to the development of postural syncope. In addition, the application of a tool capable of describing closed loop relationships could assure a more insightful characterization of the involved mechanisms. Indeed, the separation of the effects of baroreflex feedback [6] from those of the mechanical feedforward pathway [34] in the cardiovascular control loop and the disconnection of the influences of the pressure-to-flow link [74] from those of the Cushing reflex [56] in the cerebrovascular control loop might favor the comprehension of mechanisms underlying postural syncope. In addition, since the GSC operates in different frequency bands and the effect of ANS over the cardiovascular and cerebrovascular regulatory loops is frequency-dependent, the possibility of accounting for time scales might be an additional advantage.

### Limitations of the study and future developments

The present study SYNC and noSYNC groups were age- and gender-matched. Therefore, age and gender are not expected to have played a role on conclusions of the present study. However, since cardiovascular control depends on age and gender [75–78] and a relationship between cardiovascular and cerebrovascular regulations has been observed [79, 80], future studies should investigate the dependence of spectral causality indexes on gender and age. Additional reflexes known to be activated by a maneuver inducing central hypovolemia could have contributed to the conclusions of this study. Among these reflexes, we mention the sympathetic and vascular resistance arms of the baroreflex [81–83], the control of cardiac contractility and stroke volume [83–86], the dynamical response of the leg muscles to AP changes [87–90] and cardiorespiratory interactions responsible for the direct effect of respiration on HP [90–92]. Future studies should be focused on accounting for confounding factors via the acquisition of additional series and extension of GSC methods to the multivariate case [31, 32]. In the present study, we applied two portmanteau’s tests for checking the adequacy of the fitting carried out by the model. Instead of the application of portmanteau’s tests, the Durbin-Watson test might be applied. Future studies could compare the two methods for testing significance of the autocorrelation of residuals and cross-correlation between residuals to check whether discrepancies between conclusions derived from the two statistical approaches could be detected.

## Conclusions

Postural syncope propensity can be studied by applying GSC to the spontaneous fluctuations of physiological variables. The advantage of the GSC approach is to decompose in the frequency domain the strength of causal links along the two arms of the closed loop relationship. The technique was applied to the assessment of cardiovascular and cerebrovascular controls accounting for the dynamical interactions between HP and SAP and between MAP and MCBv respectively in subjects prone to develop postural syncope. Decomposition was carried out in specific frequency bands typical of the functioning of the mechanisms governing HP-SAP and MCBv-MAP variability interactions. Frequency decomposition of causal links, analysis of both the arms of the closed loop relationship, and concomitant assessment of cardiovascular and cerebrovascular controls were found to be helpful to describe the mechanisms that lead to orthostatic syncope. More specifically, we found that the postural syncope is favored by a loss of coordination between baroreflex feedback and mechanical feedforward pathway in response to HUT in the LF band and by a weaker ability of CA to limit MCBv variability driven by MAP changes at the respiratory rate during HUT. We advocate the use of the proposed approach to clarify the primary origin of postural syncope and precipitating factors, provide an early detection of people at risk of developing syncope, improve classification of patients according to the pattern of response to an imposed stressor, test the effect of possible countermeasures, and monitor the sequence of events leading to the loss of consciousness.

## Data Availability

The datasets used in the present study are available from the corresponding author on reasonable request.
